# Prediction of Ablation Volume in Percutaneous Lung Microwave Ablation: A Single Centre Retrospective Study

**DOI:** 10.3390/tomography8050206

**Published:** 2022-09-30

**Authors:** Anna Maria Ierardi, Pasquale Grillo, Maria Chiara Bonanno, Andrea Coppola, Valentina Vespro, Maria Carmela Andrisani, Davide Tosi, Paolo Mendogni, Sara Franzi, Massimo Venturini, Gianpaolo Carrafiello

**Affiliations:** 1 Department of Radiology, IRCCS Foundation Ca’ Granda Ospedale Maggiore Policlinico, 20122 Milan, Italy; 2 Postgraduate School of Diagnostic and Interventional Radiology, University of Milan, 20122 Milan, Italy; 3 Diagnostic and Interventional Radiology Unit, Ospedale di Circolo e Fondazione Macchi, ASST dei Sette Laghi, 21100 Varese, Italy; 4 Thoracic Surgery and Lung Transplantation Unit, IRCCS Foundation Ca’ Granda Ospedale Maggiore Policlinico, 20122 Milan, Italy; 5 Department of Health Sciences, University of Insubria, 21100 Varese, Italy; 6 Department of Health Sciences, University of Milan, 20122 Milan, Italy

**Keywords:** lung ablation, microwave ablation, prediction software, lung cancer treatment

## Abstract

Background: Percutaneous Microwave Ablation (MWA) of lung malignancies is a procedure with many technical challenges, among them the risk of residual disease. Recently, dedicated software able to predict the volume of the ablated area was introduced. Cone-beam computed tomography (CBCT) is the imaging guidance of choice for pulmonary ablation in our institution. The volumetric prediction software (VPS) has been installed and used in combination with CBCT to check the correct position of the device. Our study aimed to compare the results of MWA of pulmonary tumours performed using CBCT with and without VPS. Methods: We retrospectively reviewed 1-month follow-up enhanced contrast-enhanced computed tomography (CECT) scans of 10 patients who underwent ablation with the assistance of VPS (group 1) and of 10 patients who were treated without the assistance of VPS (group 2). All patients were treated for curative purposes, the maximum axial diameter of lesions ranged between 5 and 22 mm in group 1 and between 5 and 25 mm in group 2. We compared the presence of residual disease between the two groups. Results: In group 1 residual disease was seen in only 1 patient (10%) in which VPS had ensured complete coverage of the tumour. In group 2 residual disease was found in 3 patients (30%). Conclusions: Using this software during MWA of lung malignancies could improve the efficacy of the treatment compared to the conventional only CBCT guidance.

## 1. Introduction

Lung cancer is the global leading cause of death from cancer, with non-small cell lung cancer (NSCLC) being the most common primary histotype [1]. Although the standard of care for stage I NSCLC remains pneumectomy or lobectomy with lymph node sampling, up to 20% of early-stage patients are usually considered medically ineligible for surgery [2]. Moreover, while for most oncologic patients distant metastases are a sign of disease dissemination, for some tumours, such as colorectal carcinoma (CRC) and renal cell carcinoma (RCC), when the lung is the only site of disease, resection remains a viable treatment option [3,4,5]. For these reasons, in the last two decades many techniques for local disease control have been introduced, such as stereotaxic radiotherapy, radiofrequency ablation (RFA), microwave ablation (MWA) and cryoablation [6,7].

While RFA has already demonstrated similar results to surgical resection in terms of survival and disease control [8], MWA is a relatively newer technique, whose safety and efficacy for lung neoplasms was shown for the first time by Wolf et al. in 2008 [9]. Nonetheless, MWA offers some substantial advantages over RFA, such as shorter procedure time, larger and more predictable ablation volume and less intra-procedural pain [10]. Therefore, in most centres, the use of the MW technique is more common than that of RFA [11,12,13].

Anyway, none of the ablative techniques mentioned permit histopathological verification of safe margins, therefore the assessment of successful treatment relies entirely on post-procedural imaging [14] and a higher risk of residual disease has been associated with tumour size and location [9,15]. Consequently, the effort has been put into the development of software able to guide interventional radiologists in positioning the device and predicting the ablation area. A novel approach consists of the combination of volume prediction software (VPS) with Cone Beam Computed Tomography (CBCT) [16]. Using the CBCT scan obtained during the procedure, the needle/antenna can be virtually placed in the registered lung and the ablation can be simulated, visualizing possible undertreatment.

The purpose of our study was to evaluate the efficacy of this new approach, by comparing the incidence of residual disease on 1-month follow-up contrast-enhanced computed tomography (CECT) scans, after CBCT-guided MWA of primary and secondary lung tumours with and without the use of VPS.

## 2. Materials and Methods

### 2.1. Patients

Our Institutional Review Board approved this study. All patients signed informed consent for the procedure.

We retrospectively selected 20 consecutive patients who were treated for primary and secondary lung malignancies by MWA at our Institution from March 2020 to February 2022. We selected the last 10 patients who underwent ablation with the assistance of VPS (group 1) and retrieved from our archive 10 patients with similar demographical and tumour characteristics, who were treated without the software (group 2).

Indication to perform MWA was given by a multidisciplinary board composed of thoracic surgeons, oncologists, interventional and non-interventional radiologists, radiotherapists, nuclear medicine radiologists, and pneumologists, that determined the eligibility for tumour ablation on criteria such as disease stage, comorbidities, age, size, and location of the tumour following CIRSE Standard of Practice [17]. All patients were treated for curative purposes.

Both groups underwent a follow-up CECT scan 1 month after the procedure.

### 2.2. Procedure

The angiosuite was equipped with a CBCT C-arm angiograph (Azurion Clarity IQ, Philips Medical Systems, Best, The Netherlands) with a dedicated workstation running guidance and volumetric planning software (XperGuide System Philips Medical System, Best, The Netherlands).

Each patient was positioned on the angiographic table supine or prone depending on the location of the target lesion and a non-contrast-enhanced CBCT was performed. Each CBCT acquisition (XperCT, Philips Image Guided Therapy) consisted of 312 X-ray projections acquired throughout a 240-degree rotation of the C-arm around the patient in 5.2 s (120 kV, 0.4 Cu, 4 × 4 binning). After the acquisition, the X-ray projections were automatically transferred via fibre-optic connection to a dedicated 3D workstation (XtraVision, Philips Image Guided Therapy) where Feldkamp back projection was automatically performed resulting in a volumetric reconstruction with a FOV of 25 × 25 × 19 cm and 0.6 mm^3^ voxel size (matrix = 384 × 384 × 296).

**Figure 1:**
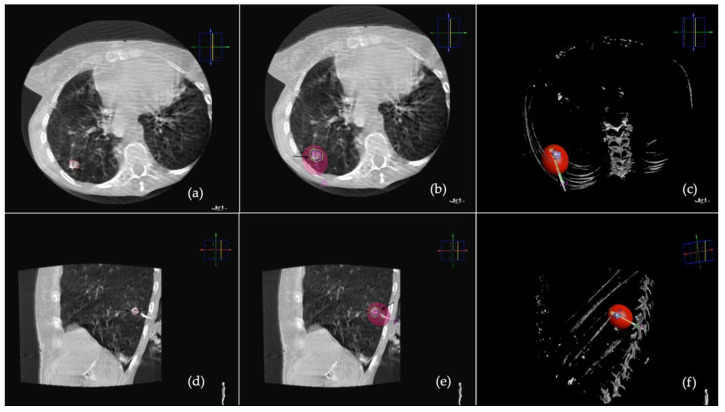
VPS was applied to CBCT images. (**a**) On axial and (**d**) sagittal CBCT scans the tumour has been segmented. (**b**) On axial and (**e**) sagittal CBCT scan, MWA is placed over the virtual one, the predicted ablation volume is seen as a purple ellipsoid and the green line (black arrow) shows that the predicted volume entirely encompasses the segmented lesion. (**c**) Axial and (**f**) sagittal 3D reconstruction of the lesion and predicted ablation volume.

**Figure 2:**
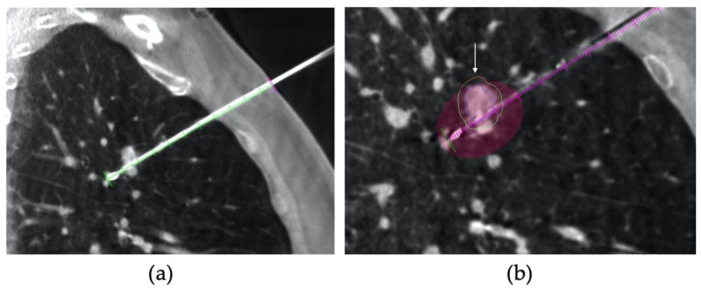
VPS was applied to CBCT images. (**a**) on sagittal CBCT scan, MWA is placed over the virtual one and (**b**) the predicted ablation volume is seen as a purple ellipsoid and the red line (white arrow) shows that the nodule is not completely encompassed in the predicted volume.

**Figure 3:**
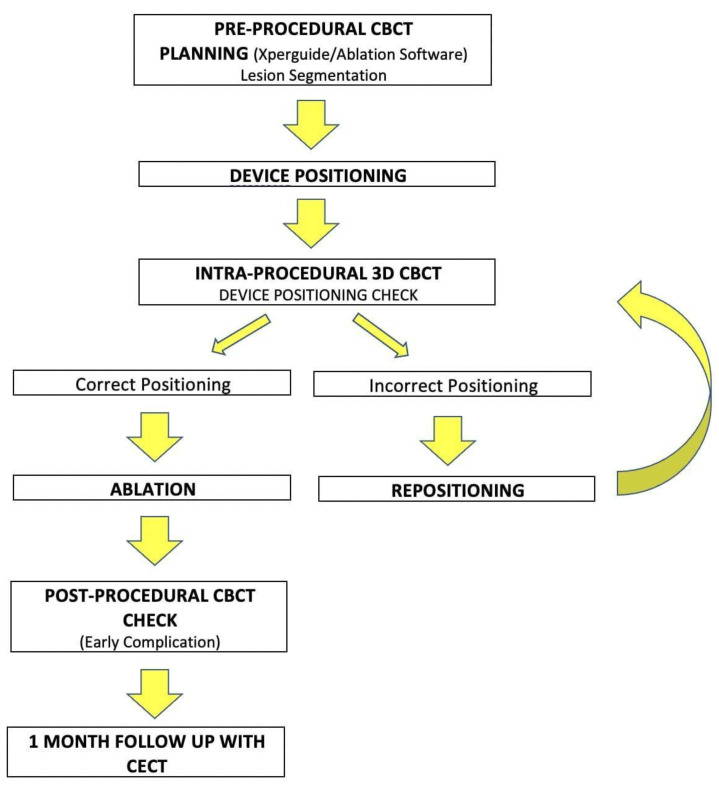
Workflow from pre-procedural CBCT planning to 1-month follow-up CECT [16]. Reproduced with the permission from Anna Maria Ierardi, Acta Radiologica, published by SAGE, 2016.

In group 1, on the first CBCT scan tumour was semi-automatically segmented using a dedicated tool (Segmentation Tool, Philips Medical Systems) to define tumour margins and volume on multiplanar reconstruction (MPR) images. Then target lesion and entry point were selected by the operator, and the system automatically calculated the trajectory by placing a virtual probe (XperGuide System Philips Medical System, Best, The Netherlands). The operator selected the desired ablation time and power, and the software generated the ablation volume based on the MWA antenna manufacturer data. The predicted volume was visualized as a purple ellipsoid on MPR images. A green line showed complete expected coverage of the lesion, as shown in Figure 1, while a red line an insufficient expected ablative margin, as shown in Figure 2. The operator had the possibility of modifying the virtual antenna position, as well as ablation time and power until the tumour was judged entirely encircled in the predicted volume. Once chosen the trajectory and ablation power and time, a straight 13.5 gauge microwave antenna (Emprint Microwave Ablation System, Medtronic, Boulder, CO, USA) was positioned over the virtual one, after performing local anaesthesia at the entrance site with 5–10 mL of Lidocaine 2%. A CBCT was then acquired to assess that the antenna was placed in the desired position, if not it was repositioned and another CBCT was performed. The process was repeated until satisfactory antenna positioning, and tumour coverage had been obtained. The steps of the procedure are summarized in Figure 3 [16].

In group 2, no segmentation of the tumour or prediction of the ablation volume was performed. The operator selected the target lesion and entry point to calculate the trajectory by placing a virtual probe; then ablation time and power were decided based on the size of the lesion. Consecutive CBCTs were performed until the antenna position was judged satisfactory by the operator.

In both groups, a post-procedural CBCT was performed to check early complications, such as pneumothorax, pleural effusion and intraparenchymal haemorrhage.

### 2.3. CT Follow-Up

Patients were studied using a multi-detector row helical 64-slice computed tomography (CT) scanner (SOMATOM Definition-Siemens Healthcare). Thoracic nonenhanced and contrast material-enhanced CT images were acquired with 3- and 1-mm collimation. Contrast-enhanced scans were acquired during the venous phase, 60 s after injection of iodinated contrast agent (Iopamiro 370, Bracco Healthcare; 1.35 mg/kg of total body weight) at an injection rate of 3 mL/s, followed by the injection of 40 mL of saline at a rate of 3 mL/s.

All CECT images were reviewed using a dedicated medical imaging workstation (Synapse PACS viewer Fujifilm Medical System USA, Inc., Miami, FL, USA).

### 2.4. Outcomes

Technical success (TS) was defined as the possibility to deploy the antenna within the target lesion, as verified by the CBCT performed before the ablation.

**Figure 4:**
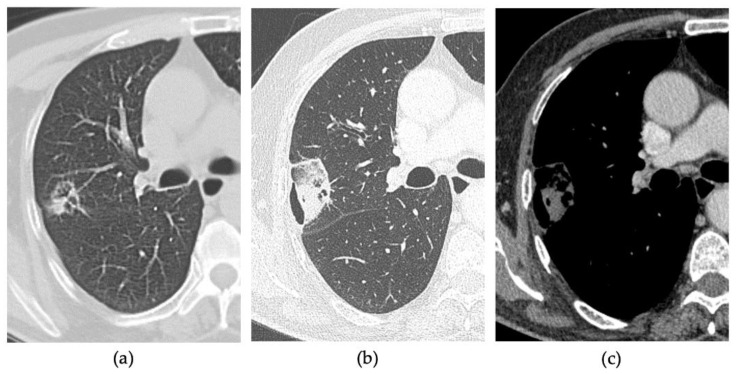
80-years-old man with lung metastasis from larynx carcinoma in the right upper lobe. (**a**) Axial pre-treatment CT. Axial 1-month follow-up CECT after MWA with VPS shows (**b**) a large consolidation with well-defined margins and cavitation and (**c**) with contrast enhancement.

**Figure 5:**
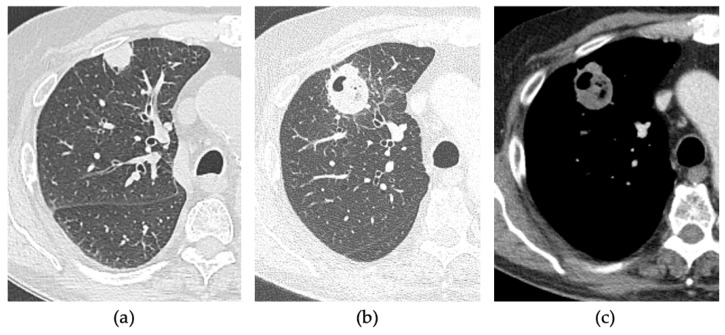
83-years-old man with NSCLC in the right upper lobe. (**a**) Axial pre-treatment CT. Axial 1-month follow-up CECT after MWA with VPS shows (**b**) a nodular-shaped margin of the consolidation of the ablation zone and (**c**) with the uptake of contrast, suggestive of residual disease.

Clinical success (CS) of the thermal ablation procedure was evaluated on CECT carried out 30 days after the procedure. The absence of enhancement within the ablated area indicated a complete ablation, as shown in Figure 4. A thin symmetric rim of peripheral enhancement (inferior than 5 mm) was considered a sign of benign peritumoral enhancement. Irregular or nodular-shaped focal soft-tissue enhancement (>15 HU) within the tumour was considered a sign of residual disease, as shown in Figure 5 [18,19,20].

Complications were graded according to the SIR (Society of Interventional Radiology) classification system [21].

Procedural time (measured from anaesthesia induction to the patient awakening), radiation exposure as dose area product (DAP) and a number of antenna repositioning were reported in both groups.

### 2.5. Statistical Analysis

Mean ± standard deviation (SD) is provided for normally distributed variables, median ± interquartile range (IQR) for non-normally distributed variables, and number and percentage for categorical variables. The Shapiro-Wilk test assessed normality. Differences between group 1 and group 2 were investigated by contingency tables and Fisher’s exact test or Pearson’s Chi-square test, when appropriate, for categorical variables, by Student’s *t*-test for continuous normally distributed variables, and by Mann-Whitney U-test for continuous non-normally distributed variables. Two-tailed tests were preferred whenever possible. *p*-values were considered significant when <0.05. SPSS v25.0.0 (IBM, Armonk, NY, USA) was used for all statistical analyses.

## 3. Results

### 3.1. Demographics and Clinical Data and Tumour Characteristics

A total of 20 patients were included in the study.

Statistical analysis of demographical and clinical data and tumour characteristics for the study population are reported in Table 1.

#### 3.1.1. Group 1

In group 1 there were 4 males (40%). The median age was 67 ± 22 years (median ± IQR; range: 57–87 years). Comorbidities were chronic obstructive pulmonary disease (COPD) (20%), coronary artery disease (CAD) (20%), arterial hypertension (40%) and kidney failure (20%).

Tumours were pathologically proven to be non-small-cell lung cancer (NSCLC) (40%) and lung metastases from colorectal carcinoma (CRC) (50%) and from renal cell carcinoma (RCC) (10%).

The mean maximal axial diameter of lesions was 12.7 ± 6.09 mm (mean ± SD; range: 5–22 mm).

#### 3.1.2. Group 2

In group 2 there were 5 males (50%). The median age was 71 ± 9.5 years (median ± IQR; range 57–80 years). Comorbidities were coronary artery disease (CAD) (40%), arterial hypertension (30%), and diabetes (30%).

Tumours were pathologically proven to be NSCLC (50%) and lung metastases from CRC (20%), from urothelial carcinoma (20%) and from larynx cancer (10%).

The mean maximal axial diameter of lesions was 15.1 ± 6.64 mm (mean ± SD; range: 5–22 mm).

### 3.2. Overall Local Tumour Control and Procedural Data

Statistical analysis of procedural and follow-up data is reported in Table 1.

#### 3.2.1. Group 1

In group 1 residual disease was seen in only 1 patient (10%) in which VPS had ensured complete coverage of the tumour.

Median DAP was 63 ± 39.45 mGy/cm^2^ (mean ± SD; range: 9.68–107 mGy/cm^2^). The mean procedure time was 33.5 ± 9.73 min (mean ± SD; range: 15–50 min). The mean fluoroscopy time was 83.9 ± 41.16 s (mean ± SD; range: 61–186). The median number of antenna repositioning was 2 ± 3 (median ± IQR; range: 0–4).

#### 3.2.2. Group 2

In group 2 residual diseases were found in 3 patients (30%).

Median DAP was 29.85 ± 23.175 mGy/cm^2^ (mean ± SD; range: 13.1–63.1 mGy/cm^2^). The mean procedure time was 34.0 ± 10.49 min (mean ± SD; range: 15–50 min). The mean fluoroscopy time was 135.8 ± 65.51 (mean ± SD; range: 62–284). The median number of antenna repositioning was 1.5 ± 2 (median ± IQR; range: 0–5).

### 3.3. Early Complications

#### 3.3.1. Group 1

There was no intraprocedural death and the overall 3-months survival rate was 100%. The complication rate was 60%. Mild PNX occurred in 3 cases (30%) and was always treated conservatively. Pleural effusion occurred in 3 cases (30%), and no treatment was needed.

#### 3.3.2. Group 2

There was no intraprocedural death and the overall 3-months survival rate was 100%. The complication rate was 60%. PNX was detected in 5 patients (50%) and an intercostal chest tube was necessary in one case (10%), judged severe. Pleural effusion occurred in 1 case (10%), and no intervention was required.

### 3.4. Statistical Analysis

In this study, we compared the incidence of residual disease on 1-month follow-up CECT, after CBCT-guided MWA of primary and secondary lung tumours with and without the use of VPS and we found no statistical difference between the two groups (10% in group 1 and 30% in group 2). We also evaluated several procedural data, such as DAP, procedure time, fluoroscopy time and the number of antenna repositioning. We observed a significant difference in fluoroscopy time (*p* = 0.048) that was shorter in group 1, while no significant difference was found in the other procedural data that were considered.

## 4. Discussion

MWA is a minimally-invasive modality for the treatment of malignant lung nodules with established efficacy and safety [9,11,12,13]. An insufficient ablation zone can lead to residual and recurrent disease, as well as overtreatment can cause tissue damage and complications [22]. In addition, the manual positioning of the probes requires a high degree of experience and can lead to multiple repositioning, therefore increasing radiation exposure for both the patient and the physician as well as the risk of complications [23]. Recently developed planning software provides the interventional radiologist with guidance on the relation between the applied energy delivery parameters, the applicator position relative to the tumour and surrounding tissues, and the anticipated treatment outcome [22,24]. Among those, the XperGuide System allows for lesion identification, antennae targeting, and look-up table-based prediction of ablation zone, representing data visually on pre- or intra-procedure CT images for visual planning. Considering these data, the radiologist can select the probe position, energy delivered and duration of ablation. The data provided by the MWA device manufacturer are based on ex vivo and in vivo experiments and, although several case series have been published, there is no statistical analysis to date that describes the clinical utility and safety of this technology [25,26].

In addition, in the literature, most reports have focused on liver ablation [27] and only a few have investigated the clinical application of such software in MWA of lung tumours [28,29,30].

Banovac et al. demonstrated the feasibility of using a pre-procedural computer-assisted volumetric planning and navigation system to deliver RFA into an artificially created lung tumour of a swine [28].

Blackmon et al. prospectively performed preoperative percutaneous MWA on fifteen patients scheduled for resection of lung nodules [29]. They compared ablation zones as measured via CT immediately after MWA versus predicted ablation zones by the Emprint software. The actual zone of ablation appeared smaller compared to the predicted one, supposedly because of the significant tissue shrinkage that occurs during MWA of the lung. On the surgical specimen, histologically complete ablation was observed in 54.5% of subjects [29].

Along with post-ablation tissue shrinkage, other factors, such as the proximity of vessels, airways, and organs, also come into play in determining the actual dimensions and shape of the ablation area. For these reasons, some software have been implemented to superimpose the theoretical ablation map on multiplanar images, combining these data with those specific to the patient. Hoffer et al. used software that simulated the effect of large blood vessel proximity and better predicted the achieved ablation volume in the in vivo models as compared to the manufacturer’s ablation maps [30].

Reisenauer et al. conducted a prospective analysis of the safety and feasibility of lung MWA using the Emprint system and observed two local tumour recurrences of the seven ablations performed [31].

Our previous study demonstrated the feasibility and safety of the VPS [16].

To our knowledge, this was the first study to compare the efficacy of MWA of lung nodules with and without the use of a VPS. The rate of residual disease that we observed in our study (10% in group 1 and 30% in group 2) is consistent with both the previous similar reports on predicted ablation volume and the other MWA lung studies in the literature, as Wolf et al. had a 26% rate [9]. We obtained the preliminary result that the predicted software might allow a lower residual disease rate (10 vs. 30%), although the difference was not statistically significant, probably due to the low number of enrolled patients and further studies need to be conducted.

In this study, we observed a significant shorter fluoroscopy time in the group where VPS was used (*p* = 0.048). However, in this group, the DAP was almost double that of Group 2, without leading to a significant difference. We suggest an explanation for this phenomenon: total DAP is given by the sum of radiation exposure caused by fluoroscopy, used for antenna positioning, and CBCT, used to evaluate the correct position of the antenna. CBCT gives a greater amount of radiation when compared to fluoroscopy. In Group 2 the operator takes more time to try to reach the tumour with the antenna, thus leading to a longer fluoroscopy time. In Group 1, the antenna is positioned with the aid of the software and fluoroscopy time is shorter, but more CBCT are performed so that the VPS can be applied to assess exactly whether the tumour area is completely covered and if not, the probe is repositioned until the entire area is included in the predicted area of ablation. Therefore, in Group 1 DAP is higher because of consecutive CBCT scans being performed even if the number of repositioning is only slightly different between the two groups (median ± IQR 2 ± 3 in Group 1 and 1.5 ± 2 in Group 2).

Our study has some limitations, firstly due to its nature of a single-centre retrospective observational study. Second, the overall number of patients was low, and for this reason, we were not able to obtain many significant results.

While a small dataset was gathered here, more robust future studies are needed to investigate the accuracy of pre-ablation planning software and its clinical utility.

## 5. Conclusions

In conclusion, this is the first study to compare the outcomes of MWA of lung tumours with and without software for the prediction of ablation volume. Our preliminary results might encourage future studies to confirm the clinical advantages of this technology.

## Figures and Tables

**Table 1:** Statistical analysis. IQR: interquartile range; SD: Standard Deviation.

Variable		Shapiro-Wilk	Group 1	Group 2	*p*-Value	Test
Sex	Male	-	4/10 (40%)	5/10 (50%)	1	Fisher
	Female	-	6/10 (60%)	5/10 (50%)		
Age		0.29	67 ± 22 (median ± IQR; range: 57–87)	71 ± 9.5 (median ± IQR; range: 57–80)	0.739	Mann-Whitney U test
Tumour	primary	-	4/10 (40%)	5/10 (50%)	1	Fisher
	metastasis	-	6/10 (60%)	5/10 (50%)		
Maximal axial diameter		0.012	12.7 ± 6.09 (mean ± SD; range: 5–22)	15.1 ± 6.64 (mean ± SD; range: 5–22)	0.411	Student’ *t*-test
DAP (mGy/cm^2^)		0.279	63 ± 39.45 (mean ± SD; range: 9.68–107)	29.85 ± 23.175 (mean ± SD; range: 13.1–63.1)	0.0089	Student’ *t*-test
Procedure Time (minutes)		0.01	33.5 ± 9.73 (mean ± SD; range: 15–50)	34 ± 10.49 (mean ± SD; range: 15–50)	0.913	Student’ *t*-test
Fluoroscopy Time (seconds)		0.001	83.9 ± 41.16 (mean ± SD; range: 61–186)	135.8 ± 65.51 (mean ± SD; range: 62–284)	0.048	Student’ *t*-test
Number of antenna repositioning		0.145	2 ± 3 (median ± IQR; range: 0–4)	1.5 ± 2 (median ± IQR; range: 0–5)	0.481	Mann-Whitney U test
Residual disease after 1 month		-	1/10 (10%)	3/10 (30%)	0.582	Fisher
Complications		-	6/10 (60%)	6/10 (60%)	1	Fisher

## Data Availability

Not applicable.
